# Lower Placental Leptin Promoter Methylation in Association with Fine Particulate Matter Air Pollution during Pregnancy and Placental Nitrosative Stress at Birth in the ENVIR*ON*AGE Cohort

**DOI:** 10.1289/EHP38

**Published:** 2016-09-13

**Authors:** Nelly D. Saenen, Karen Vrijens, Bram G. Janssen, Harry A. Roels, Kristof Y. Neven, Wim Vanden Berghe, Wilfried Gyselaers, Charlotte Vanpoucke, Wouter Lefebvre, Patrick De Boever, Tim S. Nawrot

**Affiliations:** 1 Centre for Environmental Sciences, Hasselt University, Hasselt, Belgium; 2 Louvain Centre for Toxicology and Applied Pharmacology (LTAP), Université catholique de Louvain, Brussels, Belgium; 3 Department of Biomedical Sciences, Laboratory of Protein Chemistry, Proteomics and Epigenetic Signaling (PPES), University of Antwerp, Antwerp, Belgium; 4 Biomedical Research Institute, Hasselt University, Hasselt, Belgium; 5 Department of Obstetrics, East-Limburg Hospital, Genk, Belgium; 6 Belgian Interregional Environment Agency, Brussels, Belgium; 7 Flemish Institute for Technological Research, Mol, Belgium; 8 Centre for Environment and Health, Leuven University, Leuven, Belgium

## Abstract

**Background:**

Particulate matter with a diameter ≤ 2.5 μm (PM2.5) affects human fetal development during pregnancy. Oxidative stress is a putative mechanism by which PM2.5 may exert its effects. Leptin (LEP) is an energy-regulating hormone involved in fetal growth and development.

**Objectives:**

We investigated in placental tissue whether DNA methylation of the LEP promoter is associated with PM2.5 and whether the oxidative/nitrosative stress biomarker 3-nitrotyrosine (3-NTp) is involved.

**Methods:**

LEP DNA methylation status of 361 placentas from the ENVIRONAGE birth cohort was assessed using bisulfite-PCR-pyrosequencing. Placental 3-NTp (n = 313) was determined with an ELISA assay. Daily PM2.5 exposure levels were estimated for each mother’s residence, accounting for residential mobility during pregnancy, using a spatiotemporal interpolation model.

**Results:**

After adjustment for a priori chosen covariates, placental LEP methylation was 1.4% lower (95% CI: –2.7, –0.19%) in association with an interquartile range increment (7.5 μg/m3) in second-trimester PM2.5 exposure and 0.43% lower (95% CI: –0.85, –0.02%) in association with a doubling of placental 3-NTp content.

**Conclusions:**

LEP methylation status in the placenta was negatively associated with PM2.5 exposure during the second trimester, and with placental 3-NTp, a marker of oxidative/nitrosative stress. Additional research is needed to confirm our findings and to assess whether oxidative/nitrosative stress might contribute to associations between PM2.5 and placental epigenetic events. Potential consequences for health during the neonatal period and later in life warrant further exploration.

**Citation:**

Saenen ND, Vrijens K, Janssen BG, Roels HA, Neven KY, Vanden Berghe W, Gyselaers W, Vanpoucke C, Lefebvre W, De Boever P, Nawrot TS. 2017. Lower placental leptin promoter methylation in association with fine particulate matter air pollution during pregnancy and placental nitrosative stress at birth in the ENVIRONAGE cohort. Environ Health Perspect 125:262–268; http://dx.doi.org/10.1289/EHP38

## Introduction

The *“*developmental origins of health and disease*”* concept describes how the environment may affect intrauterine development and early childhood, and how it induces developmental changes bearing long-term consequences for health and disease risk later in life (Barker 2004; Barker and Thornburg 2013). Factors such as parental lifestyle, diet, obesity, and chemical and environmental exposures have been shown to modulate disease risk (Demetriou et al. 2015; Rappaport and Smith 2010). These factors do not simply disrupt development or induce disease themselves; they can also affect onset and progress of disease development. Epigenetic events, such as changes in DNA methylation, are believed to play an important role in this process (Jaenisch and Bird 2003) and may be plausible candidates through which early-life conditions contribute to disease susceptibility later in life (Jirtle and Skinner 2007).

Exposure to ambient air pollution and particulate matter with a diameter ≤ 2.5 μm (PM_2.5_) during pregnancy may affect fetal growth and development, thereby increasing the risk of low birth weight (Ballester et al. 2010) and preterm birth (Rappazzo et al. 2014). Oxidative stress is one of the putative mechanisms by which PM_2.5_ may disrupt biological pathways/systems (Rossner et al. 2007). In addition, it has been linked with altered DNA methylation levels (Franco et al. 2008; Yara et al. 2015; Zawia et al. 2009). In biological media, an excess amount of reactive oxygen species may interact with proteins and generate 3-nitrotyrosine residues (3-NTp), a product of tyrosine nitration and a biomarker of oxidative stress and inflammation (Ischiropoulos 1998; Webster et al. 2008). Preliminary evidence showed higher expression of 3-NTp, based on immuno-histochemical staining, in high-risk pregnancies such as preeclampsia (Bosco et al. 2012) and insulin-dependent diabetes (Lyall et al. 1998). We have shown recently that the concentration of 3-NTp in the placenta is positively linked with PM_2.5_ exposure during pregnancy (Saenen et al. 2016).

The placenta is the main interface for maternal–fetal exchange of nutrients and waste, and it responds to perturbations of the maternal environment through adaptive changes (Burton and Fowden 2015; Zeltser and Leibel 2011). Recently, we reported that PM is associated with global methylation and gene-specific mitochondrial methylation in the placenta (Janssen et al. 2013, 2015) and with mitochondrial oxidative DNA damage in cord blood and maternal blood (Grevendonk et al. 2016) in the ENVIR*ON*AGE (ENVIRonmental influence *ON* early AGEing) birth cohort.

Leptin (LEP) is a hormone that regulates hunger and energy homeostasis via actions on the hypothalamus. During pregnancy, placental LEP plays a functional role in embryo implantation, intrauterine development, and fetal growth (Sagawa et al. 2002). Adverse physiological conditions during pregnancy such as maternal obesity and gestational diabetes have been associated with higher placental *LEP* methylation (Lesseur et al. 2014b), whereas other studies have found lower placental *LEP* methylation in mothers with early-onset preeclampsia (Hogg et al. 2013) or impaired glucose metabolism (Bouchard et al. 2010). Furthermore, placental *LEP* methylation was associated with significant differences in infant neurobehavior scores in boys, but there were no significant associations in girls (*n* = 223 and 221 term births, respectively) (Lesseur et al. 2014a). A possible link between PM_2.5_ exposure during pregnancy and placental *LEP* methylation has not been investigated so far. We hypothesized that gestational PM_2.5_ exposure during critical periods of prenatal life is associated with changes in placental DNA methylation of *LEP.* We also explored whether the oxidative stress biomarker 3-NTp might be acting as a mediator of the association between PM_2.5_ and *LEP* methylation by comparing the association with and without adjustment for 3-NTp.

## Methods

### Study Population

The on-going ENVIR*ON*AGE birth cohort recruits mother–newborn pairs at the delivery ward of the East-Limburg Hospital (Genk, Belgium). The hospital has a catchment area of 2,422 km^2^ and includes rural, suburban, and urban municipalities with population densities ranging from 82 to 743 inhabitants/km^2^ (FOD 2016). The participation rate of eligible mothers (mothers able to fill out a Dutch language questionnaire) in the birth cohort is approximately 61%. The questionnaire collects detailed information on maternal age, prepregnancy body mass index (BMI), maternal education and occupation, smoking status, alcohol consumption, place of residence, use of medication, parity, and ethnicity of the newborn (Janssen et al. 2015; Saenen et al. 2015). The study protocol was approved by the ethical committees of the Hasselt University and the East-Limburg Hospital, and complied with the Helsinki Declaration (http://www.wma.net/en/30publications/10policies/b3/). Written informed consent was obtained from all participants.

In the present study, 400 bio-banked placental tissue samples were randomly selected from 502 mother–newborn pairs recruited between February 2010 and May 2013. After exclusion of samples with missing data of PM_2.5_ exposure (*n* = 3) or lifestyle characteristics (*n* = 4) and those not meeting the pyrosequencing quality control criteria (*n* = 32), statistical analyses were carried out for 361 subjects in the PM_2.5_ exposure models. For the 3-NTp models, we additionally missed 3-NTp values for 48 mother–newborn pairs, resulting in 313 subjects for statistical analysis. Characteristics of these groups at enrolment were similar to those of the entire cohort (see Table S1).

### Placental Sampling

Whole placentas were stored in a –20°C freezer within 10 min after delivery. After thawing, we sampled placental tissue 1–1.5 cm below the chorioamniotic membrane to avoid membrane contamination. These biopsies were taken at a fixed location on the fetal site in the quadrant right from the main artery, approximately 4 cm away from the umbilical cord, as published previously (Janssen et al. 2014). Each biopsy was washed and rubbed thoroughly in a Petri dish filled with phosphate buffered saline to remove blood as much as possible, then snap-frozen in liquid nitrogen and archived at –80°C until DNA methylation and 3-NTp measurements.

### DNA Methylation Analysis

Genomic DNA was isolated from placental tissue samples using the QIAamp DNA mini kit (Qiagen Inc., Venlo, the Netherlands) and quantified with an ND-1000 spectrophotometer (Isogen Life Science, De Meern, the Netherlands). The DNA samples had an average yield (SD) of 8.6 (6.4) μg with an A_260/280_ ratio of 1.91 (0.08) and an A_260/230_ ratio of 2.23 (0.35). An aliquot of 500 ng DNA from each sample was sodium bisulfite–modified with the EZ-96 DNA methylation gold kit in a final elution volume of 40 μL M-elution buffer. The procedures were executed according to the manufacturer’s instructions (Zymo Research, Irvine, CA, USA). DNA methylation analysis was carried out using highly quantitative bisulfite–PCR (polymerase chain reaction) pyrosequencing. We investigated seven CpG dinucleotide sites within the promoter region of *LEP*. These sites were chosen from literature (Lesseur et al. 2013, 2014a) and data derived from the hg19 (GRCh37) UCSC Genome Browser (http://genome.ucsc.edu/) (Kent et al. 2002; Rosenbloom et al. 2015), illustrating significant transcription factor binding by ChIP analysis to the CpG island promoter region of interest. Figure S1 displays the chromosomal position of the *LEP* promoter region investigated. PCR and sequencing primers were designed with the Pyromark Assay Design software (forward primer: 5´-AGG​TGT​ATA​TTG​AGG​GTT​TAG​GGT​TAG-3´; biotinylated reverse primer: 5´-ACA​TCC​CTC​CTA​ACT​CAA​TTT​C-3´; and sequencing primer: 5´-GGG​AGT​TGG​AGT​TAG​AAA​TG-3´). The PCR product of the *LEP* region of interest was amplified from bisulfite-modified DNA with the Pyromark PCR kit (Qiagen, Inc.). Cycling conditions started with an initial PCR activation at 95°C for 15 min, followed by 45 cycles at 94°C for 30 sec, 56°C for 30 sec, and 72°C for 30 sec, to end with a final extension for 10 min at 72°C. The PCR product was sequenced with a Pyromark Q24 Instrument (Qiagen Inc.). We excluded 32 samples that did not pass the standard quality control implemented in the Pyromark Q24 Advanced software (Qiagen Inc.) from further analysis. The percentage of methylation was determined with the Pyromark Q24 Advanced software. The software used different parameters for quality assessment including unsuccessful bisulfite treatment (allowed percentage), peak height threshold (required peak height), and stringency levels (pattern/sum deviation in variable positions). The efficiency of the bisulfite-conversion process was assessed using non-CpG cytosine residues within the sequence. Duplicates of the pyrosequencing runs (*n* = 38) were highly correlated for the mean of the CpG sites (*r* ^2^ = 0.99) as well as for each CpG site separately (*r* ^2^ ranging from 0.90 to 0.99).

### 3-Nitrotyrosine Protein Measurement

Thawed placental tissue samples with a wet weight of approximately 10 mg were manually homogenized on ice in lysis buffer [10 mM Tris-HCl (pH 7.4), 150 mM NaCl, 1% Triton X-100 and Protease Inhibitor Cocktail, Complete, mini, (Roche, Basel, Switzerland)] and sonicated three times in bursts of 10 sec. The samples were allowed to settle for 20 min on ice and then centrifuged at 16,000 × *g* for 20 min at 4°C. The supernatants were aliquoted and frozen at –20°C until further measurements.

Total protein concentration of the placenta sample was determined with the Bio-rad protein assay according to the manufacturer’s instructions (Bio-rad, Nazareth, Belgium). The amount of 3-NTp in each sample was quantified with a competitive ELISA (Oxiselect nitrotyrosine ELISA kit; Cell Biolabs, San Diego, CA, USA) and absorbance measurements were performed at 450 nm using a FLUOstar Omega (BMG Labtech, Offenburg, Germany). Concentrations of 3-NTp were determined using a standard curve of predetermined nitrated BSA (bovine serum albumin) standards. Data were normalized to the amount of protein present in the sample and were presented as nM/mg protein.

### Particulate Matter Air Pollution Exposure

PM_2.5_ exposure (μg/m^3^) concentrations were modeled using a spatial temporal interpolation method (kriging) (Janssen et al. 2008) for each mother’s residential address in combination with a dispersion model. The interpolation method uses land-cover data obtained from satellite images (CORINE land-cover data set; http://www.eea.europa.eu/data-and-maps/data/corine-land-cover-2006-clc2006-100-m-version-12-2009) and pollution data collected from a governmental stationary monitoring network (http://www.irceline.be/). Coupled with a dispersion model (Lefebvre et al. 2013; Maiheu et al. 2013) that uses emissions from point sources and line sources, this model chain provides PM_2.5_ values in a high-resolution receptor grid (average grids of 25 × 25 m). Overall model performance was evaluated by leave-one-out cross-validation including 34 monitoring points for PM_2.5_. Validation statistics of the interpolation tool explained > 80% of the temporal and spatial variability in the Flemish Region of Belgium (Maiheu et al. 2013). To explore potentially critical exposure windows, we averaged the daily interpolated PM_2.5_ concentrations for each of the three pregnancy trimesters, i.e., first trimester (week 1–13), second trimester (week 14–26) and third trimester (week 27–delivery). The date of conception was estimated on the basis of the first day of the mother’s last menstrual period, combined with the first ultrasound exam. Complete information for the residential address during pregnancy was obtained by questionnaire and checked with hospital records. For those who moved during pregnancy, we calculated the trimester-specific exposures allowing for the changes in address during this period (based on the daily exposure levels at the different residential addresses).

### Statistical Analyses

Statistical analyses were carried out using SAS software (version 9.3; SAS Institute Inc., Cary, NC, USA). Continuous data were presented as mean ± SD and categorical data as frequencies and percentages. The 3-NTp content was log_10_-transformed to normalize the distribution. To avoid multiple testing, we evaluated the association between the placental methylation status of the *LEP* promoter region of interest and gestational PM_2.5_ exposure or placental 3-nitrotyrosine content using mixed-effects models. In these models, the seven studied CpG sites were integrated into a single factor (individual CpG sites treated as repeated measures using an unstructured covariance structure model) (Janssen et al. 2015). For each trimester-specific PM_2.5_ exposure model, we adjusted for *a priori* chosen covariates including as continuous variables maternal age, gestational age, and prepregnancy body mass index (BMI); and as categorical variables, newborn sex (boy, girl), maternal education (low, middle, high), smoking status (never smoker, former smoker, smoker), ethnicity of the newborn (non-European, European origin), and trimester-specific season (season at gestational exposure window: autumn, winter, spring, summer). Socioeconomic status was based upon the mothers’ education and coded as “low” (no diploma or primary school), “middle” (high school), or “high” (college or university degree). Smoking status was defined as never smoker, former smoker (quit smoking before pregnancy), and smoker (continued smoking during pregnancy). The ethnicity of the newborn was defined on the basis of the native country of the newborn’s grandparents and was classified “of European origin” when two or more grandparents were European. In addition, because placental *LEP* methylation was measured at birth, we mutually adjusted each model for the other gestational exposure windows to estimate the independent effect of each trimester of exposure. The results are presented for each gestational exposure window as an absolute percentage change in placental *LEP* methylation for a trimester-specific interquartile range (IQR) increment in PM_2.5_ (μg/m^3^). The 3-NTp models were adjusted for the aforementioned covariates, except for trimester-specific season which was replaced by season at delivery, and the estimated effect sizes are presented for a doubling in placental 3-NTp content (nM/mg protein). *p*-Value < 0.05 was used to define statistical significance.

In a sensitivity analysis, we examined the associations between placental *LEP* methylation and PM_2.5_ exposure or placental 3-NTp content while excluding mothers with gestational diabetes, gestational hypertension, preeclampsia, or preterm births. Furthermore, additional adjustment of the main model for mother’s total weight gain was evaluated. We also examined the associations between the methylation at individual CpG sites and PM_2.5_ exposure or placental 3-NTp using multiple linear regressions (see Figure S2 and Table S2). Finally, we included placental 3-NTp as a covariate in the mixed-effects model of the association between placental *LEP* methylation and trimester-specific PM_2.5_ to determine whether estimated associations changed with adjustment for this potential mediator.

## Results

### Study Population Characteristics and Measurements in Placenta

Demographic, lifestyle, and other characteristics of the total group of 361 mother–newborn pairs (mean maternal age, 29.4 ± 4.7 years) are presented in Table 1. Pregestational BMI averaged 24.1 ± 4.3, and 52.4% of the mothers had obtained a higher education degree. Fifty mothers (13.9%) reported having smoked during pregnancy, whereas the majority (67.3%) never smoked cigarettes. The newborn population, comprising 189 boys (52.3%), had a mean gestational age of 39.3 weeks (range, 35–42). Most of the newborns were term-born infants (96.1%) and the majority were primiparous (51.2%) or secundiparous (37.7%) births. Mean birth weight and length were 3,426 ± 450 g and 50.5 ± 2.1 cm respectively. The population characteristics of the 3-NTp group (*n* = 313) were consistent with those from the total group (Table 1). The 3-NTp levels averaged (range) 3,703 (100–23,681) nM/mg protein and the mean (range) methylation levels of the seven CpG sites investigated in the placental *LEP* promoter region are shown in Table 2. The mean methylation level of CpG4 (61.5%) was substantially higher than the other six CpG sites (< 22.3%).

**Table 1:** Characteristics of mother–newborn pairs.

Characteristics	Total group (*n *= 361)	3-NTp group (*n *= 313)
Mother
Age, years	29.4 ± 4.7	29.5 ± 4.6
Prepregnancy BMI (kg/m^2^)	24.1 ± 4.3	24.1 ± 4.5
Total weight gain (kg)^*a*^	14.8 ± 6.9	14.6 ± 7.1
Education
Low	47 (13.0)	38 (12.1)
Middle	125 (34.6)	105 (33.6)
High	189 (52.4)	170 (54.3)
Self-reported smoking status
Never smoker	243 (67.3)	212 (67.7)
Former smoker	68 (18.8)	59 (18.9)
Smoker	50 (13.9)	42 (13.4)
Parity
1	185 (51.2)	164 (52.4)
2	136 (37.7)	116 (36.1)
≥ 3	40 (11.1)	33 (10.5)
Pregnancy complications
Gestational diabetes	13 (3.6)	13 (4.2)
Gestational hypertension	7 (1.9)	6 (1.9)
Preeclampsia	2 (0.6)	2 (0.6)
Preterm birth	14 (3.9)	12 (3.8)
Newborn
Sex
Male	189 (52.3)	164 (52.4)
Ethnicity
European	310 (85.9)	271 (86.6)
Gestational age (weeks)	39.3 ± 1.3	39.3 ± 1.3
Born at term (≥ 37 weeks)	347 (96.1)	301 (96.2)
Season of delivery
Spring	100 (27.7)	88 (28.1)
Summer	51 (14.1)	41 (13.1)
Autumn	102 (28.3)	86 (27.5)
Winter	108 (29.9)	98 (31.3)
Apgar score after 5 min
6	1 (0.3)	0 (0)
7	6 (1.7)	6 (1.9)
8	16 (4.4)	15 (4.8)
9	102 (28.2)	86 (27.5)
10	236 (65.4)	206 (65.8)
Birth weight (g)	3,426 ± 450	3,424 ± 450
Birth length (cm)^*a*^	50.5 ± 2.1	50.5 ± 2.1
3-NTp, 3-nitrotyrosine. Continuous data are presented as mean ± SD; categorical variables as *n* (%). ^***a***^Data available for 360 and 312 subjects respectively.		

**Table 2:** Molecular measurements on placental tissue samples (*n *= 361).

Measurement	Mean (range)
3-NTp (nM/mg protein)^*a*^	3,703 (100–23,681)
*LEP* methylation (%)
CpG1	10.0 (0.53–42.9)
CpG2	12.7 (0.66–38.0)
CpG3	8.5 (0.91–34.9)
CpG4	61.5 (33.5–88.6)
CpG5	13.6 (2.0–34.9)
CpG6	13.5 (1.1–38.2)
CpG7	22.3 (0.52–47.6)
^***a***^3-Nitrotyrosine, geometric mean (range), *n *= 313.	

### PM_2.5_ Exposure

The distributions of the outdoor PM_2.5_ levels for the different time windows of pregnancy are shown in Table 3. The average (25th–75th percentile) trimester-specific PM_2.5_ exposure was 15.7 (11.5–19.7) μg/m^3^ for the first trimester, 15.5 (11.4–18.9) μg/m^3^ for the second trimester, and 17.2 (12.0–21.9) μg/m^3^ for the third trimester of pregnancy.

**Table 3:** Exposure characteristics of airborne particulate matter ≤ 2.5 (PM_2.5_) (*n *= 361).

Time windows PM_2.5_ (μg/m^3^)	Mean ± SD	10th percentile	25th percentile	Median	75th percentile	90th percentile	IQR
Trimester 1 (1–13 weeks)	15.7 ± 5.3	10.0	11.5	13.9	19.7	24.0	8.2
Trimester 2 (14–26 weeks)	15.5 ± 4.9	10.0	11.4	14.6	18.9	22.9	7.5
Trimester 3 (27 weeks–delivery)	17.2 ± 5.8	10.1	12.0	16.9	21.9	25.6	9.9

### Placental *LEP* Promoter Methylation at Birth and its Association with PM_2.5_ Exposure or Placental 3-Nitrotyrosine

**Figure 1:**
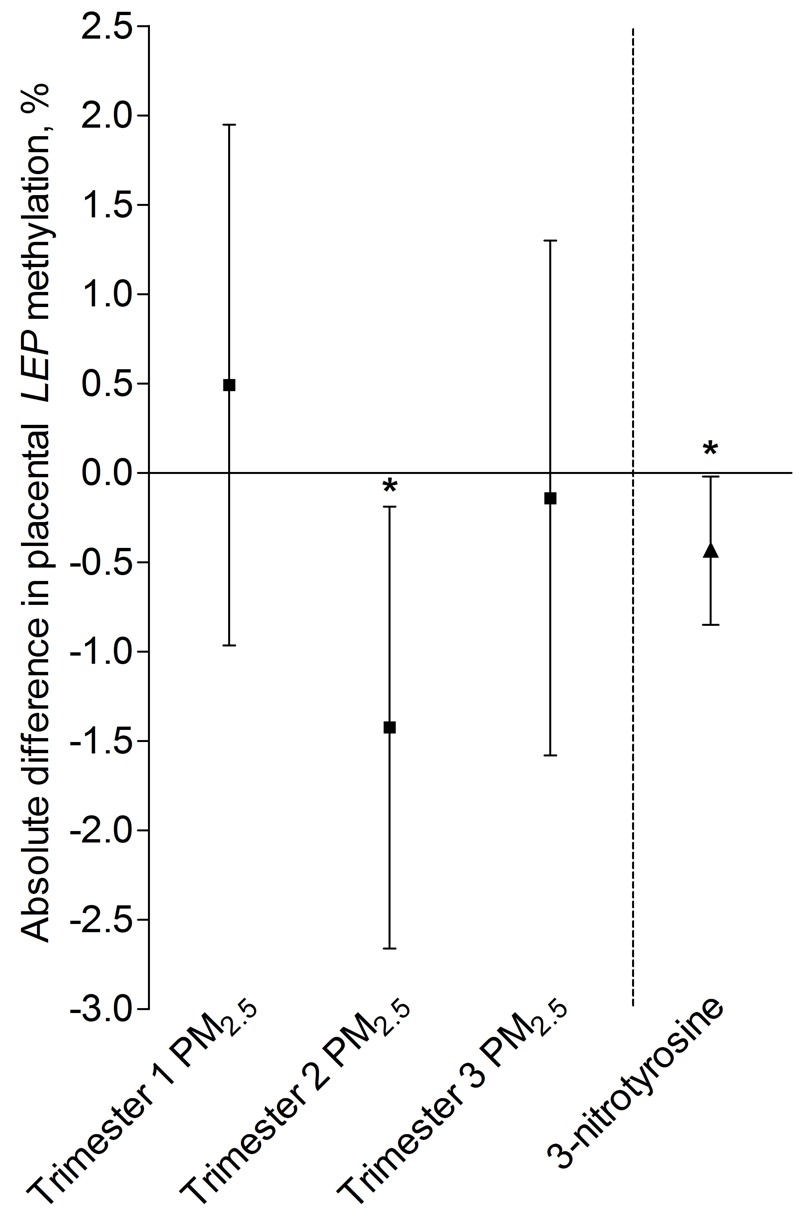
Placental *LEP* promoter DNA methylation in association with PM_2.5_ exposure for different time windows of pregnancy (*n *= 361) or placental 3-nitrotyrosine (3-NTp) at birth (*n *= 313). Models were adjusted for newborn sex, maternal age, maternal education, maternal smoking status, gestational age, prepregnancy BMI, ethnicity, and season (i.e., gestational trimester-specific season in the PM_2.5_ exposure models and season of delivery in the 3-NTp model). The trimester-specific PM_2.5_ exposure models were mutually adjusted for the other gestational exposure windows to estimate the independent effect of each trimester of exposure. Estimates are presented as an absolute percentage difference in placental *LEP* promoter DNA methylation for a trimester-specific interquartile range increment in PM_2.5_ exposure (trimester 1: 8.2 μg/m^3^; trimester 2: 7.5 μg/m^3^; trimester 3: 9.9 μg/m^3^) or a doubling in 3-NTp content (nM/mg protein). *p < 0.05.

The seven CpG sites investigated in the placental *LEP* promoter region were highly correlated with each other (*r* = 0.47–0.88). In male neonate placentas, the *LEP* promoter methylation was higher compared to placentas of female neonates [1.33%; 95% confidence interval (CI): 0.40, 2.27%, *p* = 0.005 for the male neonate placenta vs. the female neonate placenta]. *LEP* promoter methylation was not associated with mother’s prepregnancy BMI (0.003%; 95% CI: –0.10, 0.11%; *p* = 0.96 for a 1-unit increase in BMI based on the adjusted mixed-effects model of *LEP* methylation and trimester-specific PM_2.5_) (see Table S2) or total weight gain (–0.042%; 95% CI: –0.11, 0.03%; *p* = 0.24 for a 1-unit increase in total weight gain based on the same model, but without adjustment for pre-pregnancy BMI) (see Table S2). We fitted a mixed-effects model to evaluate the association between the methylation levels in the *LEP* promoter region of interest (individual CpG sites treated as repeated measures) and PM_2.5_ exposure. After adjustment for newborn sex, maternal age, maternal education, smoking status, gestational age, prepregnancy BMI, ethnicity, and gestational trimester-specific season, we estimated that overall *LEP* methylation in the placenta was 1.4% lower (95% CI: –2.7, –0.19%, *p* = 0.02) with an IQR increment in second-trimester PM_2.5_ exposure (7.5 μg/m^3^) (Figure 1). No associations were observed between overall *LEP* methylation and an IQR increment in first-trimester PM_2.5_ exposure (8.2 μg/m^3^) (0.49%; 95% CI: –0.97, 1.95%; *p* = 0.51) or third-trimester PM_2.5_ exposure (9.9 μg/m^3^) (–0.14%; 95% CI: –1.58, 1.30%, *p* = 0.13).

Both before (data not shown) and after adjustment for covariates (newborn sex, maternal age, maternal education, smoking status, gestational age, prepregnancy BMI, ethnicity, and season of delivery), a doubling in placental 3-NTp content at birth was associated with a significantly lower overall methylation level of the *LEP* region evaluated (–0.43%; 95% CI: –0.85, –0.02%, *p* = 0.04) (Figure 1).

### Sensitivity Analysis

**Figure 2:**
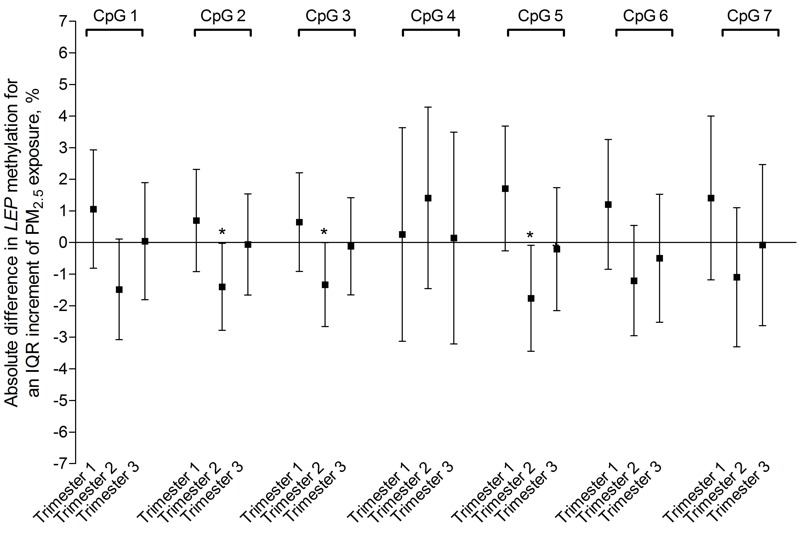
Placental CpG-specific *LEP* promoter DNA methylation in association with PM_2.5_ exposure for different time windows of pregnancy (*n *= 361). Models were adjusted for newborn sex, maternal age, maternal education, maternal smoking status, gestational age, prepregnancy BMI, ethnicity, and gestational trimester-specific season. The trimester-specific PM_2.5_ exposure models were mutually adjusted for the other gestational exposure windows to estimate the independent effect of each trimester of exposure. Estimates are presented as absolute percentage difference in *LEP* promoter DNA methylation for a trimester-specific interquartile range increment in PM_2.5_ exposure (trimester 1: 8.2 μg/m^3^; trimester 2: 7.5 μg/m^3^; trimester 3: 9.9 μg/m^3^). *p < 0.05.

A sensitivity analysis in which preterm births (*n* = 14 for total group; *n* = 12 for 3-NTp group), mothers with gestational diabetes/hypertension (*n* = 20 for total group; *n* = 19 for 3-NTp group), and mothers with preeclampsia (*n* = 2 for both groups) were excluded showed very little change in the estimated associations between the overall placental methylation of the *LEP* promoter region and second-trimester PM_2.5_ exposure or placental 3-NTp content (see Table S3). Additional adjustment of the main mixed-effects model for mother’s total weight gain during pregnancy did not change statistical significance (see Table S3). Evaluation of the individual CpG sites based on multiple linear regression models suggested that associations were strongest with four of the seven individual CpG sites (CpG1: –1.5%; 95% CI: –3.1, 0.10%; *p* = 0.06; CpG2: –1.4%; 95% CI: –2.8, –0.03%, *p* = 0.05; CpG3: –1.3%; 95% CI: –2.7, –0.008%, *p* = 0.05; and CpG5: –1.8%; 95% CI: –3.4, –0.09%, *p* = 0.04) (Figure 2). For a doubling in placental 3-NTp, the results of *LEP* methylation suggested solid associations with two individual CpG sites (CpG2: –0.50%; 95% CI: –0.97, –0.03%, *p* = 0.04 and CpG5: –0.53%; 95% CI: –1.10, –0.05%, *p* = 0.07) (see Figure S2). Finally, adjustment of the main mixed-effects model for placental 3-NTp content (*n* = 313) resulted in a weakening of the association between placental *LEP* promoter methylation and PM_2.5_ exposure of the second gestational window (–1.1%; 95% CI: –2.4, 0.22%; *p* = 0.10 vs. –1.33%; 95% CI: –2.63, –0.03%, *p* = 0.04)_._

## Discussion

The human placenta is the anatomo-physiological barrier between mother and fetus. External factors may interfere with placental functions and alter signaling pathways, hormone production, nutrient and waste transfer, embryo implantation, and cellular growth (Myllynen et al. 2005). Literature suggests that epigenetic mechanisms play a role in the complex interplay between environment and genes, and may predispose to disease phenotypes (Jaenisch and Bird 2003; Jirtle and Skinner 2007). In a previous study on the ENVIR*ON*AGE birth cohort we showed a positive association between PM_2.5_ exposure and placental 3-NTp (Saenen et al. 2016). The key findings of the present study are the significant inverse associations of both second-trimester PM_2.5_ exposure and placental 3-NTp concentrations at birth with DNA methylation of the *LEP* promoter region in the placenta. Associations varied among the individual CpG sites.

During pregnancy, LEP is thought to play a functional role in embryo implantation, intrauterine development, and fetal growth (Hassink et al. 1997; Sagawa et al. 2002). It has been shown that umbilical cord blood LEP concentrations were positively correlated with term birth weight in a study population that included 70 newborns with intrauterine growth retardation and 62 newborns classified as having normal growth (Jaquet et al. 1998). In the placenta, LEP is synthesized by trophoblasts and mostly secreted in the maternal blood circulation (Sagawa et al. 2002). Studies indicated that the contribution of placental LEP secretion to circulating fetal leptin is minimal (Lepercq et al. 2001; Linnemann et al. 2001), and that fetal adipose tissue is most likely the main source of fetal LEP (Clapp and Kiess 1998; Jaquet et al. 1998; Lepercq et al. 2001).

Reproductive events involving leptin are crucial for adequate functional development of the placenta, including regulation of nutrient transport, placental angiogenesis, trophoblast mitogenesis, and immunomodulation (Henson and Castracane 2000). When LEP binds to its receptor, it stimulates angiogenic factors such as the vascular endothelial growth factor, thereby activating p38, MAPK, and Akt pathways that induce proliferation, motility, and angiogenesis (Garonna et al. 2011). These processes are critical in placental development, angiogenesis in villi, and fetal-derived vascularization (Demir et al. 1997). Furthermore, *in situ* hybridization and immunohistochemistry of placental tissue showed that placental LEP in humans is expressed in syncytiotrophoblast cells (facing maternal circulation) and villous vascular endothelial cells (facing fetal circulation) (Lea et al. 2000).

For the second-trimester exposure window, we found a decreased *LEP* methylation in placental tissue at the fetal side in association with an IQR increment (7.5 μg/m^3^) in PM_2.5_ exposure. The negative association with placental *LEP* methylation is in line with evidence of *LEP* hypomethylation in placenta of complicated pregnancies such as early-onset preeclampsia (Hogg et al. 2013) and impaired glucose metabolism (Bouchard et al. 2010), both known to adversely influence placental growth and vascularization. This is consistent with earlier observations of increased placental LEP expression reported in other studies of complicated pregnancies (Lepercq et al. 1998; Mise et al. 1998). Placental LEP is believed to exert a local protective immunomodulating response (Ashworth et al. 2000). Because successful pregnancies are associated with downregulation of proinflammatory cytokines such as tumor necrosis factor alpha, LEP may have a local protective response at the maternal–fetal interface (Lea et al. 1997; Takahashi et al. 1999). In the context of this literature evidence, future studies should address the consequences of hypomethylation of the placental *LEP* status and its possible involvement in placental immunomodulation and vascularization.

In addition to the negative association between placental *LEP* promoter methylation and mid-gestation PM_2.5_ exposure, we found also a negative association between *LEP* promoter methylation and placental 3-NTp content, which was independent of maternal smoking and other factors. The prevalence of 3-NTp, based on immuno-histochemical staining, has been observed in two small studies with different high-risk pregnancies, including preeclampsia and gestational diabetes (Bosco et al. 2012; Lyall et al. 1998). In these complicated pregnancies, the higher presence and level of 3-NTp residues in placental tissue may indicate vascular damage (Myatt and Cui 2004). An experimental study investigating diesel exhaust particle (DEP) exposure in mice suggested that *in utero* DEP promotes vascular oxidative stress as shown by elevated 3-NTp protein modification (Weldy et al. 2014). The presence of 3-NTp in placenta and its association with PM_2.5_ exposure (Saenen et al. 2016) may be indicative of a PM-linked inflammation.

It is important to mention that a TATA box and a potential binding site for the C/EBP transcription factor are present in the studied promotor region. CpG4 is situated in the recognition sequence of C/EBP (Marchi et al. 2011). An experimental study investigating methylation-dependent transcriptional activity of a human *LEP* promoter fragment in Lisa-2 cultured cells (a liposarcoma cell line) showed that methylation of the CpG4 site (corresponding to CpG position –51 in Figure 6 of Melzner et al. 2002) was important for down-regulation of promoter activity of *LEP* (Melzner et al. 2002). Demethylation of the CpG sites, which are proximal to the TATA box, was found essential for *LEP* expression in primary fibroblasts and HeLa cells (Marchi et al. 2011). PM_2.5_ air pollution was not significantly associated with methylation of the CpG4 site in our study. We observed that the individual CpG sites varied in average methylation, especially at the CpG4 site, which was substantially higher methylated than the other CpG sites. Methylation at the CpG2, 3, and 5 sites, which flank the transcription factor sequence as well as the TATA box region, was significantly lower in association with an IQR increase in second-trimester PM_2.5_ air pollution.

We acknowledge some study limitations. First, pyrosequencing assays can capture only a small region of 80 base pairs in the *LEP* promoter region, and it is possible that we missed additional methylation changes in the promoter region. On the other hand, bisulfite-PCR-pyrosequencing has the advantage of being a highly standardized quantitative procedure that allowed us to obtain accurate results (Dejeux et al. 2009; Tost and Gut 2007). Second, the placenta is a tissue of different cell types with the presence of maternal and cord blood. Because the composition of placenta samples can differ and might influence DNA methylation and gene expression patterns, a standardized methodological protocol was used for sampling each placenta at an almost identical position. Furthermore, maternal and cord blood was removed as much as possible, and the placental 3-NTp content was expressed per mg of placental protein. Third, we cannot exclude any residual confounding by other environmental factors or characteristics associated with the exposures and outcome. Despite the fact that we used a high-resolution receptor grid to estimate PM_2.5_ exposure, there is still a possibility for exposure misclassification.

## Conclusions

We estimated significant negative associations of placental *LEP* promoter region methylation with PM_2.5_ exposure during the second gestational trimester, and with placental 3-NTp, a marker of oxidative/nitrosative stress, at birth. The associated CpG methylation sites are flanking a nucleotide sequence with a regulatory function (Marchi et al. 2011). Additional research is needed to confirm our findings in other study populations and evaluate the potential impact of placenta *LEP* methylation on health during the neonatal period and later in life.

## Supplemental Material

(691 KB) PDF
