# Supplementary material for: Lower Placental Leptin Promoter Methylation in Association with Fine Particulate Matter Air Pollution during Pregnancy and Placental Nitrosative Stress at Birth in the ENVIRONAGE Cohort
Source: Environ Health Perspect. 2016 Sep 13;125(2):262–8. doi: 10.1289/EHP38 (PMC5289914; doi:10.1289/EHP38)
Supplement: (691 KB) PDF [file EHP38.s001.acco.pdf]

Environ Health Perspect
DOI: 10.1289/EHP38

Note to readers with disabilities: *EHP* strives to ensure that all journal content is accessible to all readers. However, some figures and Supplemental Material published in *EHP* articles may not conform to 508 standards due to the complexity of the information being presented. If you need assistance accessing journal content, please contact ehponline@niehs.nih.gov. Our staff will work with you to assess and meet your accessibility needs within 3 working days.

Supplemental Material

Lower Placental Leptin Promoter Methylation in Association with Fine Particulate Matter Air Pollution during Pregnancy and Placental Nitrosative Stress at Birth in the ENVIR*ON*AGE Cohort

Nelly D. Saenen, Karen Vrijens, Bram G. Janssen, Harry A. Roels, Kristof Y. Neven, Wim Vanden Berghe, Wilfried Gyselaers, Charlotte Vanpoucke, Wouter Lefebvre, Patrick De Boever, and Tim S. Nawrot

Table of Contents

Table S1: Characteristics of the groups in the study compared to the entire cohort at the moment of selection (ENVIR*ON*AGE birth cohort)....................2
Table S2: Effect estimates of the covariates in the models associating placental *LEP* methylation with gestational trimester-specific $PM_{2.5}$ exposure or placental 3-nitrotyrosine at birth. 3
Table S3. Associations between placental CpG-specific *LEP* methylation and placental 3-nitrotyrosine at birth. 4
Figure S1: Region of interest in the *LEP* promoter based upon Assembly GRCh37/hg19 of the UCSC genome browser (Kent et al. 2002; Rosenbloom et al. 2015)**.** 5
Figure S2: Associations between placental CpG-specific *LEP* methylation and placental 3-nitrotyrosine at birth. Estimates are adjusted for newborn sex, maternal age, maternal education, smoking status, pre-pregnancy BMI, gestational age, ethnicity, and season of delivery. Results are presented as an absolute percentage difference in *LEP* promoter DNA methylation for a doubling in placental 3-nitrotyrosine content (nM/mg protein)....................6

Table S1: Characteristics of the groups in the study compared to the entire cohort at the moment of selection (ENVIR*ON*AGE birth cohort).

Characteristics	Total group (n = 361)	3-nitrotyrosine group (n = 313)	Entire cohort (n=502)
**Mother**			
Age, y	29.4 ± 4.7	29.5 ± 4.6	29.2 ± 4.6
Pre-pregnancy BMI	24.1 ± 4.3	24.1 ± 4.5	24.3 ± 4.6
Education			
Low	47 (13.0%)	38 (12.1%)	60 (12.0%)
Middle	125 (34.6%)	105 (33.6%)	179 (35.8%)
High	189 (52.4%)	170 (54.3%)	261 (52.2%)
Self-reported smoking status			
Never-smoker	243 (67.3%)	212 (67.7%)	326 (64.9%)
Past-smoker	68 (18.8%)	59 (18.9%)	99 (19.7%)
Smoker	50 (13.9%)	42 (13.4%)	77 (15.3%)
Parity			
1	185 (51.2%)	164 (52.4%)	266 (53.0%)
2	136 (37.7%)	116 (36.1%)	182 (36.3%)
≥ 3	40 (11.1%)	33 (10.5%)	54 (10.7%)
**Newborn**			
Sex			
Male	189 (52.3%)	164 (52.4%)	253 (50.4%)
Ethnicity			
European	310 (85.9%)	271 (86.6%)	432 (86.2%)
Gestational age, w	39.3 ± 1.3	39.3 ± 1.3	39.3 ± 1.3
Born at term (≥ 37 w)	347 (96.1%)	301 (96.2%)	487 (97.0%)
Season of delivery			
Spring	100 (27.7%)	88 (28.1%)	135 (26.9%)
Summer	51 (14.1%)	41 (13.1%)	86 (17.1%)
Autumn	102 (28.3%)	86 (27.5%)	135 (26.9%)
Winter	108 (29.9%)	98 (31.3%)	146 (29.1%)
Apgar score after 5 min			
6	1 (0.3%)	0 (0%)	1 (0.2%)
7	6 (1.7%)	6 (1.9%)	7 (1.4%)
8	16 (4.4%)	15 (4.8%)	20 (4.0%)
9	102 (28.2%)	86 (27.5%)	148 (29.5%)
10	236 (65.4%)	206 (65.8%)	326 (64.9%)
Birth weight, g	3426 ± 450	3424 ± 450	3443 ± 438
Birth length, cm[a]	50.5 ± 2.1	50.5 ± 2.1	50.4 ± 1.9

Continuous data are presented as mean ± SD; categorical variables as number (%).
[a] Data available for 360, 312, and 501 subjects respectively.

Table S2: Effect estimates of the covariates in the models associating placental *LEP* methylation with gestational trimester-specific $PM_{2.5}$ exposure or placental 3-nitrotyrosine at birth.

Co-variates	Trimester-specific $PM_{2.5}$-exposure model		3-nitrotyrosine model	
	β (95% CI)	p-value	β (95% CI)	p-value
**Mother**				
Age, y	-0.077 (-0.18, 0.03)	0.14	-0.092 (-0.20, 0.02)	0.10
Pre-pregnancy BMI	0.003 (-0.10, 0.11)	0.96	-0.015 (-0.12, 0.09	0.78
Total weight gain	-0.042 (-0.11, 0.03)	0.24	-0.043 (-0.11, 0.03)	0.22
Gestational age, d	-0.032 (-0.08, 0.02)	0.22	-0.032 (-1.03, 1.22)	0.26
Education				
Low	0.015 (-1.52, 1.55)	0.98	0.59 (-1.04, 2.22)	0.47
Middle	-0.12 (-1.20, 0.96)	0.83	0.09 (-1.03, 1.22)	0.87
High	Ref.			
Self-reported smoking status				
Never-smoker	0.67 (-0.76, 2.10)	0.36	1.06 (-0.45, 2.57)	0.17
Past-smoker	0.57 (-1.11, 2.25)	0.51	1.51 (-0.26, 3.28)	0.09
Smoker	Ref.		Ref.	
**Newborn**				
Sex				
Male	1.33 (0.40, 2.27)	0.005	1.30 (0.32, 2.27)	0.01
Female	Ref.		Ref.	
Ethnicity				
European	0.81 (-0.57, 2.19)	0.25	1.23 (-0.23, 2.70)	0.10
non-European	Ref.		Ref.	
Season				
Autumn	1.31 (-0.62, 3.24)	0.18	-1.37 (-2.67, -0.077)	0.04
Winter	-1.15 (-4.03, 1.74)	0.43	-0.49 (-1.96, 0.99)	0.52
Spring	0.35 (-1.57, 2.26)	0.72	-1.26 (-2.73, 0.22)	0.09
Summer	Ref.		Ref.	

Effect estimates are presented as an absolute percentage difference in *LEP* methylation for a unit increase in the corresponding covariate. The trimester-specific $PM_{2.5}$-exposure model included all covariates shown in the table including all trimester-specific $PM_{2.5}$ exposures and trimester-specific season, except for total weight gain. The same covariates were used in the 3-nitrotyrosine model including $\log_{10}$ 3-NTp and season at delivery. Similar models were used to estimate total weight gain, excluding pre-pregnancy BMI.

Table S3. Sensitivity analysis: associations between placental *LEP* methylation and trimester-specific $PM_{2.5}$ exposure or 3-NTp at birth with exclusion of pregnancy complications or additional adjustment for total weight gain.

	Trimester 1 $PM_{2.5}$ exposure		Trimester 2 $PM_{2.5}$ exposure		Trimester 3 $PM_{2.5}$ exposure		3-NTp	
	%	95% CI	%	95% CI	%	95% CI	%	95% CI
**Main analysis**	0.49	(-0.97, 1.95)	-1.42	(-2.66, -0.19)	-0.14	-1.58, 1.30	-0.48	(-0.88, -0.09)
- preterm births	0.43	(-0.97, 1.87)	-1.51	(-2.74, -0.27)	-0.58	-2.03, 0.87	-0.50	(-0.91, -0.09)
- gestational diabetes	0.54	(-0.97, 2.04)	-1.37	(-2.64, -0.11)	-0.15	-1.62, 1.33	-0.38	(-0.80, 0.04)
- hypertension	0.44	(-0.97, 1.90)	-1.43	(-2.67, -0.18)	0.06	-1.40, 1.52	-0.40	(-0.82, 0.03)
- pre-eclamspia	0.55	(-0.97, 2.00)	-1.41	(-2.65, -0.18)	-0.12	-1.55, 1.32	-0.42	(-0.83, -0.002)
+ total weight gain	0.54	(-0.97,2.00)	-1.40	(-2.64, 0.16)	-0.13	-1.57, 1.31	-0.43	(-0.84, -0.02)

Models were adjusted for newborn sex, maternal age, maternal education, maternal smoking status, gestational age, pre-pregnancy BMI, ethnicity, and season; i.e. gestational trimester-specific season in the $PM_{2.5}$ exposure models and season of delivery in the 3-NTp model. The trimester-specific $PM_{2.5}$ exposure models were mutually adjusted for the other gestational exposure windows to estimate the independent effect of each trimester of exposure. Estimates are presented as an absolute percentage difference in placental *LEP* promoter DNA methylation for a trimester-specific interquartile range increment in $PM_{2.5}$ exposure (trimester 1: 8.2 $\mu g/m^3$; trimester 2: 7.5 $\mu g/m^3$; trimester 3: 9.9 $\mu g/m^3$) or a doubling in 3-NTp content (nM/mg protein).

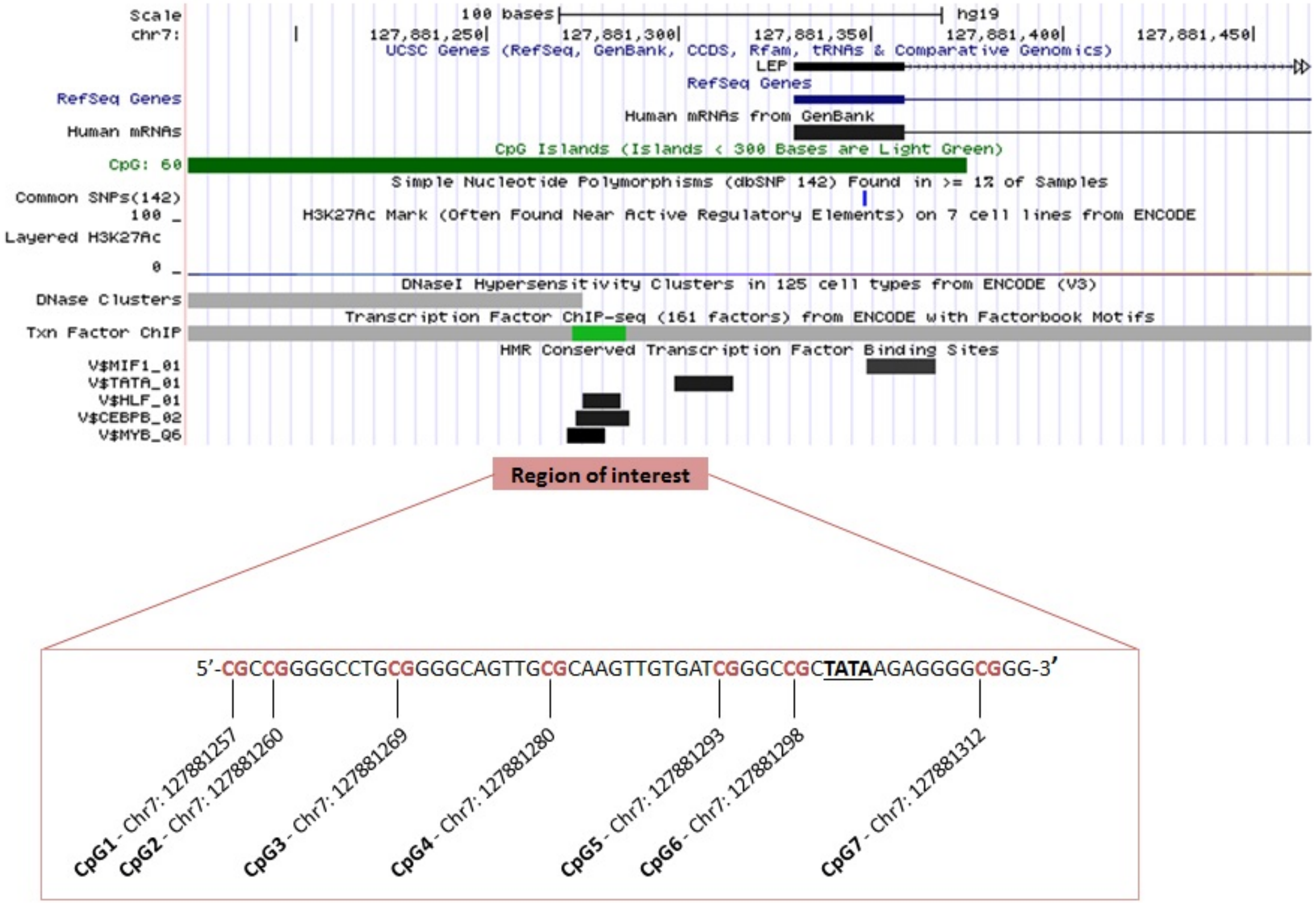

Figure S1: Region of interest in the *LEP* promoter based upon Assembly GRCh37/hg19 of the UCSC genome browser (Kent et al. 2002; Rosenbloom et al. 2015).

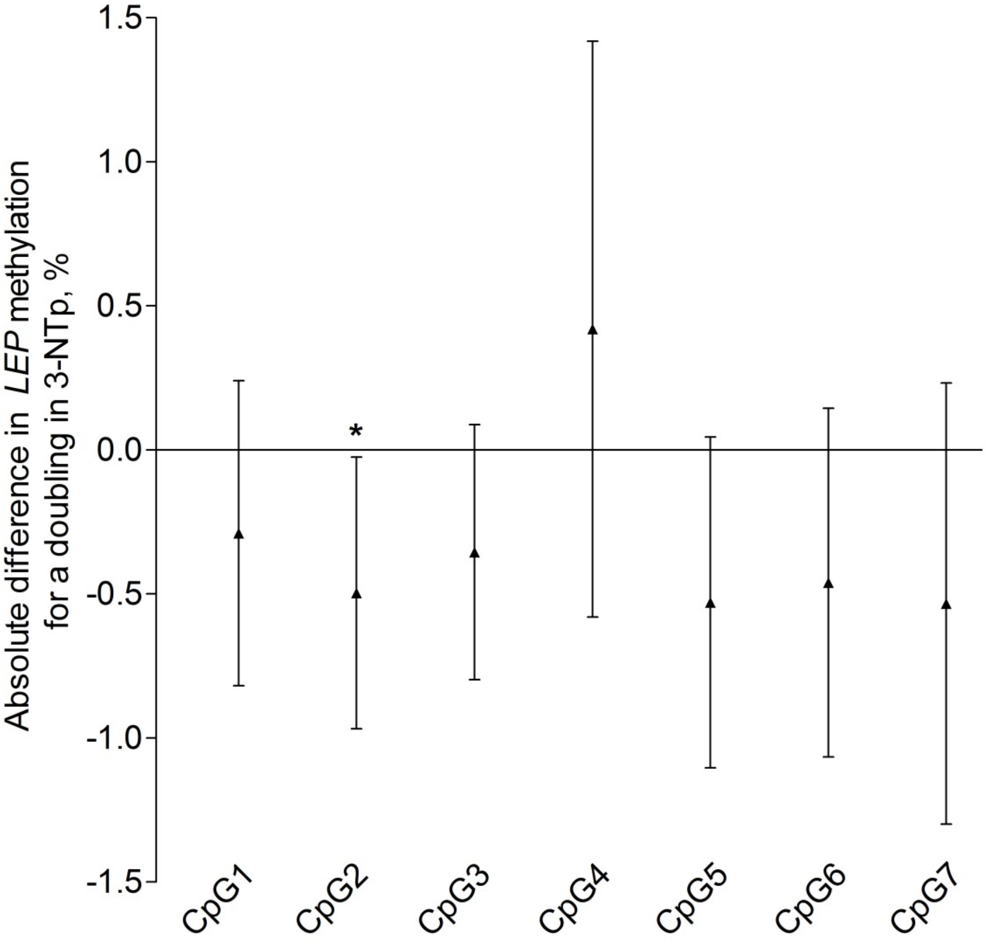

Figure S2: Associations between placental CpG-specific *LEP* methylation and placental 3-nitrotyrosine at birth. Estimates are adjusted for newborn sex, maternal age, maternal education, smoking status, pre-pregnancy BMI, gestational age, ethnicity, and season of delivery. Results are presented as an absolute percentage difference in *LEP* promoter DNA methylation for a doubling in placental 3-nitrotyrosine content (nM/mg protein). * $p<0.05$
